# Cellular Glucose Transport and Inflammatory Blood Markers in Rheumatoid Arthritis Spectrum: An Immunometabolic Perspective

**DOI:** 10.7759/cureus.110719

**Published:** 2026-06-12

**Authors:** Diptimayee Upadhyay, Subhashree Ray, Bandana Thakur, Rajesh Kumar Bhola, Chetan Kumar Kellellu

**Affiliations:** 1 Department of Biochemistry, Institute of Medical Sciences (IMS) and Sum Hospital, Siksha ‘O’ Anusandhan (Deemed to be University), Bhubaneswar, IND; 2 Department of Pathology, Institute of Medical Sciences (IMS) and Sum Hospital, Siksha ‘O’ Anusandhan (Deemed to be University), Bhubaneswar, IND

**Keywords:** autoimmune synovial disease, glut1, hk2, immunometabolism, rheumatoid arthritis

## Abstract

Background: Rheumatoid arthritis (RA) is increasingly recognized as an immunometabolic disorder. This study evaluated whether glucose-handling markers, including glucose transporter 1 (GLUT1) and hexokinase 2 (HK2), and circulating inflammatory markers differed between RA cases and healthy controls and whether these markers were associated with DAS28-based disease activity in an exploratory secondary analysis.

Methods: A retrospective secondary analysis was conducted using data from 160 participants, comprising 80 cases and 80 controls. Variables included demographic characteristics, fasting glucose, erythrocyte sedimentation rate (ESR), C-reactive protein (CRP), interleukin-6 (IL-6), serum amyloid A (SAA), neutrophil-to-lymphocyte ratio (NLR), platelet-to-lymphocyte ratio (PLR), Disease Activity Score in 28 joints (DAS28), GLUT1, and HK2. Nonparametric tests were used for group comparisons, and Spearman's correlation was used to assess within-case associations.

Results: Cases demonstrated significantly higher GLUT1 and HK2 levels than controls (both p < 0.001), along with elevated ESR, interleukin-6, SAA, NLR, and PLR. HK2 was significantly higher in high-activity than moderate-activity cases (p = 0.037), whereas GLUT1 was not. Within-case correlations were weak and nonsignificant.

Conclusion: These findings support a measurable immunometabolic signature in RA. HK2 may show exploratory association with DAS28-based activity categories; however, prospective validation is required before clinical application.

## Introduction

Rheumatoid arthritis (RA) is a chronic immune-mediated inflammatory arthritis characterized by persistent synovial inflammation, immune dysregulation, and progressive joint damage [1,2]. While in RA, the involvement of autoimmunity and inflammatory destruction of the joints has been the hallmark, emerging data suggest that there is a more complex model in play, where also dysfunction of immune cells, activation of stromal cells, and alterations in cellular metabolism play a role in maintaining disease activity [1,3]. The clinical relevance is that a partial reduction or not consistent changes in the conventional blood inflammatory markers can result in an inflammatory burden at the tissue level [1,2].

In addition to being a by-product of systemic inflammation, the synovium is a pathological compartment that also contains fibroblast-like synoviocytes (FLS), macrophages, lymphocytes, endothelial cells, and soluble inflammatory mediators [2,4]. In this new microenvironment, FLS turn into an apoptotic-resistant, cytokine-producing, and invasive phenotype that results in the formation of pannus, cartilage destruction, and local joint destruction [2,4]. The inflammatory niche can be hidden peripherally by reciprocal communications between immune cells and resident stromal cells that foster a self-perpetuating inflammatory niche [1,4].

Increased use of the glycolytic pathway is one of the major mechanisms that leads to immune activation in the synovial compartment and to the subsequent development of continuous synovial disease [5,6]. Increased glucose uptake and glycolytic flux in activated immune cells and RA-FLS, which subsequently result in enhanced proliferation, production of cytokines, migration to the joints, matrix remodeling, and survival in the hypoxic inflammatory environment [3,5]. Changes will enable synovial cells to adapt to energy and biosynthetic demands in chronic inflammation and correlate metabolic adaptation with joint structural damage [3,6]. In experimental studies, the biological plausibility of glucose-handling pathways as disease-relevant markers is demonstrated, and targeting glycolytic pathways has been shown to inhibit inflammatory and invasive functions of RA-FLS [5,6].

The glycolytic mediators, such as glucose transporter 1 (GLUT1) and hexokinase 2 (HK2), are a few that are significant in RA [7,8]. In metabolically active inflammatory cells, GLUT1 will be upregulated, leading to increased glucose import and the increased GLUT1 will lead to increased glucose import in those cells, which will be utilized for glycolytic metabolism in the cells [5,8]. Especially relevant is the fact that it is HK2, which is downstream of the glucose uptake and can therefore be a measure of continued glycolytic engagement, rather than only glucose uptake [7,8]. The expression level of HK2 has been shown to mirror changes in disease activity and is potentially diagnostic and has a disease severity relevance in RA and could thus serve as a biomarker of immunometabolic disease activity [7,8].

While conventional inflammatory markers still play a key role in the evaluation of RA, these markers do not completely reflect the underlying biology of the disease [9-11]. Commonly used two markers for the acute phase are erythrocyte sedimentation rate (ESR) and C-reactive protein (CRP), which have different performance in various states of treatment and patients [10,11] and are used for clinical interpretation of inflammatory activity. Furthermore, the acute phase signaling pathways of interleukin-6 (IL-6) and systemic inflammation are involved in RA, as well as the serum amyloid A (SAA), which can be used as an indicator for inflammatory burden in some clinical contexts [12,13]. Other readily available hematological markers of systemic inflammation and disease activity (the neutrophil-to-lymphocyte (NLR) and platelet-to-lymphocyte (PLR) ratios) were also investigated [11].

The various multiscore measures of disease activity used in the assessment of RA are referred to as "composite" disease activity measures [10]. The Disease Activity Score in 28 joints (DAS28) is a score that includes the joint examination, patient assessment, and the acute-phase response and is used to indicate the activity of the disease and to be used when interpreting this [10]. However, the metabolism markers and circulating inflammatory markers might not move hand in hand, especially in the context of drug administration, disease duration, platform used for the assays, time of specimen collection, and the comorbid metabolic status and heterogeneity of the data sets, for which there is currently little knowledge [1,9]. If a secondary data analysis is conducted, this is especially important because the biomarkers may have been collected in a nonstandardized way, and the clinical data may have been collected in a nonstandardized way, before the analysis [9,10].

In the present study, we analyzed a de-identified secondary dataset of patients with RA and healthy controls to evaluate immunometabolic and inflammatory differences between groups. The primary objective was to compare GLUT1 and HK2 expression between RA cases and healthy controls. Secondary objectives were to compare circulating inflammatory markers between groups, examine GLUT1 and HK2 across DAS28-based disease activity categories, and assess exploratory correlations between metabolic markers, inflammatory indices, fasting glucose, and DAS28 within the RA cohort.

## Materials and methods

Study design and setting

This study was a secondary analysis of a fully de-identified institutional dataset compiled from records collected between January 1995 and March 2026 at IMS & SUM Hospital, Bhubaneswar, India. The data were selected for the study based on clinical, biochemical, hematologic, inflammatory, and immunometabolic parameters relevant to RA. The dataset was obtained in anonymized form from an institutional research repository and was originally generated from routine clinical and laboratory records before de-identification. No participant recruitment, direct patient contact, specimen collection, or additional laboratory testing was performed for the present analysis.

Because the dataset covered an extended period, information regarding changes in laboratory methods, assay platforms, calibration procedures, specimen timing, treatment exposure, and clinical management over time was not available. These factors were therefore considered limitations of the secondary analysis. Analysis was conducted to assess differences between disease and control groups in inflammatory markers and glucose-handling markers and to examine their relationship with disease activity categories.

Study population

A total of 160 participants, including 80 patients with RA and 80 healthy controls, were included in the study. Diagnoses were previously reported using the 2010 American College of Rheumatology/European League Against Rheumatism classification criteria, with a minimum disease duration of six months. Controls were clinically healthy individuals without any reported chronic inflammatory, autoimmune, or metabolic disorders. Controls were selected from the available de-identified dataset and were not individually matched to cases by age or sex. Therefore, baseline age and sex differences were considered when interpreting between-group comparisons. Disease activity was categorized according to the DAS28 scores available in the dataset. Moderate disease activity was defined as DAS28 >3.2 to ≤5.1, and high disease activity was defined as DAS28 >5.1, consistent with established DAS28 interpretation thresholds and the DAS28 originally described and validated by Prevoo et al. [14].

Inclusion criteria

Selection criteria for cases were age 18 years or older, documentation of a diagnosis of RA, and disease duration of six months or more. Controls were adult individuals who did not have any known autoimmune, chronic inflammatory, or metabolic disease and were clinically normal at the time of data collection.

Exclusion criteria

Participants were excluded based on dataset annotations if they had a known diagnosis of diabetes mellitus, other known metabolic disorders, or were known to be taking medication that would significantly affect glucose metabolism. Detailed treatment-exposure data were not available in the de-identified dataset and could not be incorporated into the analysis. Records that could not be interpreted reliably were excluded from the relevant analysis.

Use variables and data handling

The dataset contained age, sex, disease duration, BMI, FPG, ESR, CRP, IL-6, SAA, NLR, PLR, DAS28, disease activity category, GLUT1 expression, and HK2 expression. Before analysis, the dataset was reviewed for de-identification, variable availability, completeness, coding consistency, plausible numeric ranges, unit consistency, duplicate records, and alignment of participant-level values across columns.

Records with incomplete information for a specific analysis were excluded only from that analysis. No imputation procedures were performed because the dataset was analyzed in its original de-identified form. Data completeness was considered acceptable for all variables included in the final analyses. One serology-related variable was excluded before analysis because the column was misaligned relative to the remaining dataset structure, preventing reliable linkage of values to individual participants. Because data integrity could not be verified for this variable, it was removed and was not included in any statistical analysis. DAS28 was available as a continuous variable, and the disease activity category was used for subgroup analysis.

Statistical analysis

Medians (with interquartile ranges) were used to summarize continuous variables, and reported frequencies and percentages were used to summarize categorical variables. Several distributions of biomarker data were not normal, and metadata for harmonization of the assays were not available, so nonparametric methods were employed. The Mann-Whitney U test was applied to compare the continuous data between the cases and controls, and Fisher's exact test was applied to compare the categorical data between cases and controls. The Mann-Whitney U test was used to compare the moderate activity and high activity cases. Spearman's rank correlation was used to determine the association between GLUT1, HK2, inflammatory indices, fasting glucose, and DAS28. The significance of the results was determined at p < 0.05. Because the dataset was retrospective and de-identified, multivariable adjustment for age, sex, BMI, fasting glucose, treatment exposure, and other potential confounders was not performed. The findings should therefore be interpreted as exploratory unadjusted associations. Adjustment for multiple comparisons was not performed because this was an exploratory, hypothesis-generating secondary analysis; consequently, the results require cautious interpretation and prospective confirmation.

Ethical considerations

This study involved secondary analysis of a fully de-identified dataset. No patient contact, intervention, or access to identifiable private information occurred during the present analysis. All analyses were performed using anonymized data, and no attempt was made to identify individual participants or link data to personal identifiers. The study was conducted in accordance with the principles of the Declaration of Helsinki, and institutional ethics approval details are provided in the Disclosures section.

## Results

Cohort characteristics

There were 160 participants (80 cases, 80 controls). There was a higher percentage of female participants in the case group compared with the control group (87.5% vs 61.3%, p < 0.001). The mean age of the cases was significantly younger than that of the controls (45.00 years vs 56.00 years; p < 0.001). BMI was significantly higher in cases than in controls (p = 0.026), and the fasting glucose level was significantly different between cases and controls (p < 0.001) (Table 1).

**Table 1:** Case-control comparison of demographic, inflammatory, and metabolic variables ESR: erythrocyte sedimentation rate; CRP: C-reactive protein; IL-6: interleukin-6; GLUT1: glucose transporter 1; HK2: hexokinase 2 Values are presented as median (interquartile range) unless otherwise stated. Statistical comparisons were performed using the Mann-Whitney U test for continuous variables and Fisher’s exact test for categorical variables, which indicates statistically significant values (p < 0.05)

Variable	Case	Control	Test statistic	p-value
Sample size, n	80	80	-	-
Female sex, n (%)	70 (87.5)	49 (61.3)	Fisher exact = 3.16	0.002*
Age, years	45.00 (40.00-55.00)	56.00 (46.50-65.00)	U = 1973.5	<0.001*
Body mass index, kg/m²	26.74 (25.45-30.39)	26.25 (22.90-28.82)	U = 3852.5	0.026*
Fasting glucose, mg/dL	86.50 (77.50-92.00)	111.00 (92.00-126.00)	U = 1079.5	<0.001*
ESR, mm/hr	90.00 (84.50-99.00)	25.00 (14.00-54.25)	U = 6040.5	<0.001*
CRP, mg/L	0.69 (0.33-1.70)	2.70 (1.90-3.70)	U = 923.0	<0.001*
IL-6, pg/mL	27.45 (21.80-33.75)	5.30 (4.33-6.88)	U = 6400.0	<0.001*
Serum amyloid A, mg/L	14.50 (12.07-38.08)	5.25 (3.70-8.18)	U = 5582.0	<0.001*
Neutrophil-lymphocyte ratio	4.50 (3.90-5.43)	2.10 (1.50-4.12)	U = 5221.0	<0.001*
Platelet-lymphocyte ratio	189.82 (166.07-212.26)	135.00 (113.75-174.00)	U = 5000.0	<0.001*
GLUT1 expression	3.15 (2.60-3.80)	1.40 (1.10-1.62)	U = 6400.0	<0.001*
HK2 expression	3.20 (2.70-3.50)	1.30 (1.10-1.50)	U = 6400.0	<0.001*

CRP values were reported in mg/L as recorded in the source dataset. The CRP result was discordant with the other inflammatory markers, as cases showed lower CRP despite higher ESR, IL-6, SAA, NLR, and PLR. Therefore, the CRP finding was retained as recorded but interpreted cautiously in view of possible source-dataset, assay, treatment, or timing-related factors.

Inflammatory and metabolic comparisons between cases and controls

All cases were significantly higher than controls for the ESR, IL-6, SAA, NLR, PLR, and expression of HK2 (p < 0.001). Among the immunometabolic panel, GLUT1 and HK2 stood out. The level of CRP was only reduced in cases, while the others were increased, which was deemed a "discordant" result and was noted with the proviso that it may be affected by assay, treatment, or dataset.

Disease activity subgroup analysis

Of the 80 cases, 44 were considered to have moderate disease activity, and 36 were considered to have high disease activity. The scores of DAS28 were significantly higher in the high-activity group than in the moderate-activity group (5.85 vs 4.16, p < 0.001), indicating a proper separation of the subgroups. There was a significant difference between the expression of HK2 in high activity cases and moderate activity cases (p = 0.037), while the expression of GLUT1 was nonsignificant (p = 0.176). There were no significant differences between the activity groups for the other inflammatory and hematologic markers (Table 2). This finding may reflect overlapping systemic inflammatory profiles between moderate- and high-activity groups and the composite structure of DAS28, which is not determined by inflammatory markers alone.

**Table 2:** Comparison of moderate- versus high-activity cases ESR: erythrocyte sedimentation rate; CRP: C-reactive protein; IL-6: interleukin-6; DAS28: Disease Activity Score in 28 joints; GLUT1: glucose transporter 1; HK2: hexokinase 2 Values are presented as median (interquartile range). Statistical comparisons were performed using the Mann-Whitney U test, which indicates statistically significant values (p < 0.05)

Variable	Moderate (n = 44)	High (n = 36)	Test statistic	p-value
Age, years	45.00 (39.75-53.00)	48.00 (40.75-60.00)	U = 678.5	0.273
Disease duration, years	4.60 (3.00-6.20)	5.85 (3.00-6.20)	U = 710.0	0.428
Body mass index, kg/m²	26.74 (25.43-30.39)	26.74 (25.45-29.66)	U = 793.0	0.996
Fasting glucose, mg/dL	89.00 (77.50-92.00)	80.00 (77.00-92.75)	U = 838.0	0.659
ESR, mm/hr	90.00 (82.50-102.50)	90.00 (86.75-98.00)	U = 788.0	0.973
CRP, mg/L	0.68 (0.33-1.70)	0.70 (0.37-1.53)	U = 792.0	1.000
IL-6, pg/mL	27.30 (20.55-33.92)	27.45 (23.27-33.60)	U = 772.0	0.850
Serum amyloid A, mg/L	14.50 (8.35-37.47)	17.40 (12.80-40.50)	U = 732.5	0.568
Neutrophil-lymphocyte ratio	4.35 (3.88-5.53)	4.70 (3.90-5.33)	U = 766.0	0.805
Platelet-lymphocyte ratio	189.82 (166.29-213.87)	189.40 (164.85-212.09)	U = 847.0	0.598
DAS28 score	4.16 (3.73-4.67)	5.85 (5.50-6.29)	U = 0.0	<0.001*
GLUT1 expression	3.00 (2.50-3.70)	3.20 (2.80-3.80)	U = 652.0	0.176
HK2 expression	2.85 (2.50-3.30)	3.30 (3.20-3.80)	U = 576.0	0.037*

Correlation analysis

Spearman's rank correlation analysis was used to assess the relationship between the immunometabolic markers GLUT1 and HK2 and other clinical, biochemical, and inflammatory parameters in the case cohort. There was no statistically significant correlation between GLUT1, HK2, and ESR, CRP, IL-6, SAA, NLR, PLR, fasting glucose, or DAS28 score. There was no statistically significant correlation between GLUT1, HK2, and ESR, CRP, IL-6, SAA, NLR, PLR, fasting glucose, and DAS28 score. The correlation coefficient and p-values for each variable are shown in Table 3.

**Table 3:** Spearman correlations between metabolic markers and inflammatory indices in cases ESR: erythrocyte sedimentation rate; CRP: C-reactive protein; IL-6: interleukin-6; GLUT1: glucose transporter 1; HK2: hexokinase 2 Spearman's rank correlation analysis was used to calculate correlation coefficients and p-values. Indicates statistically significant values (p < 0.05)

Variable	GLUT1 rho	GLUT1 p-value	HK2 rho	HK2 p-value
ESR, mm/hr	0.03	0.804	-0.03	0.797
CRP, mg/L	0.08	0.501	-0.05	0.660
IL-6, pg/mL	0.16	0.159	-0.02	0.838
Serum amyloid A, mg/L	0.02	0.891	-0.05	0.663
Neutrophil-lymphocyte ratio	-0.05	0.676	0.06	0.607
Platelet-lymphocyte ratio	0.08	0.498	0.16	0.169
Fasting glucose, mg/dL	-0.10	0.368	-0.03	0.781
DAS28 score	0.14	0.409	0.13	0.423

**Figure 1:**
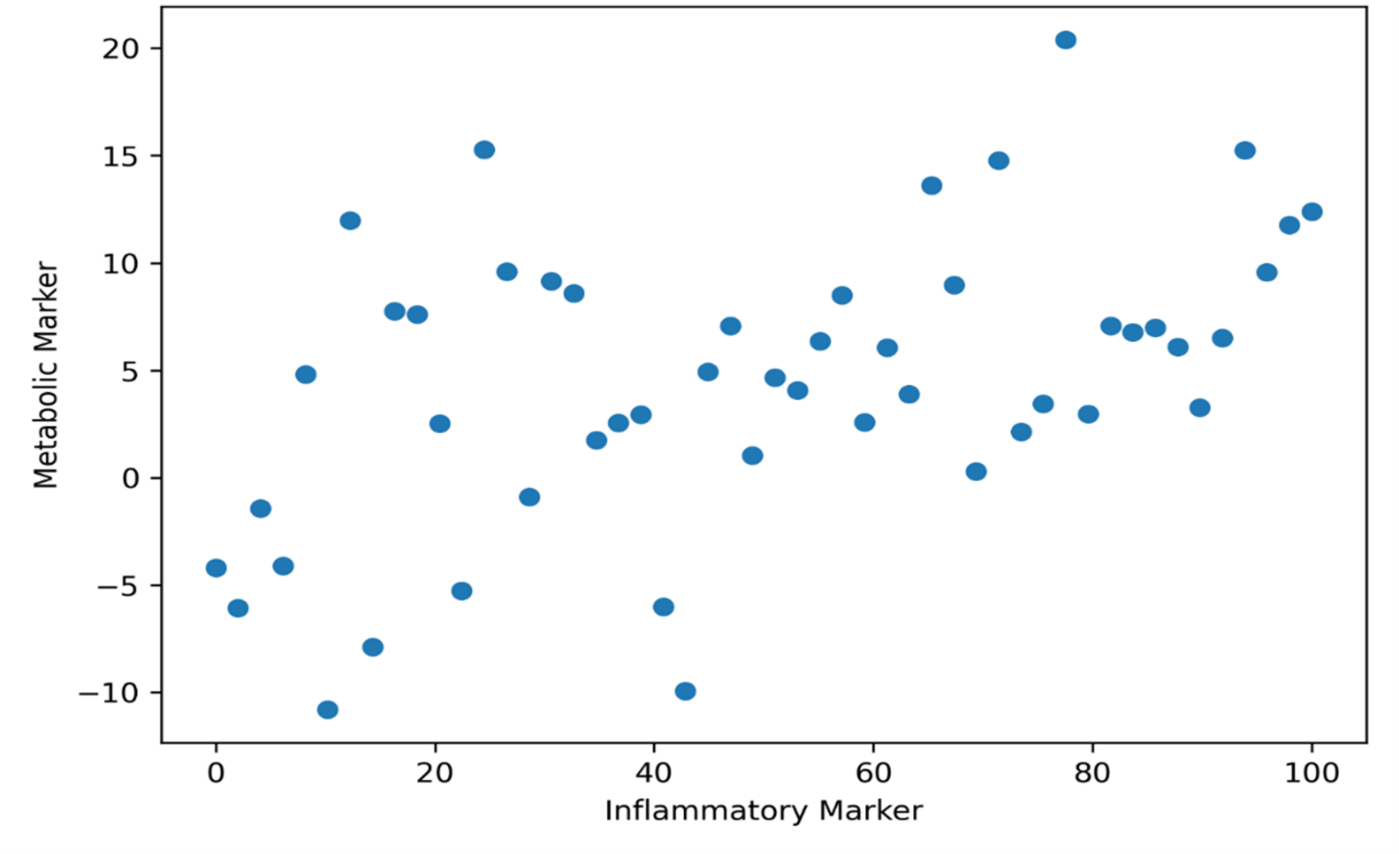
Correlation of metabolic markers with inflammatory activity

Scatter plot analysis further demonstrated weak and nonsignificant relationships between metabolic markers and selected inflammatory indices (Figure 1).

**Figure 2:**
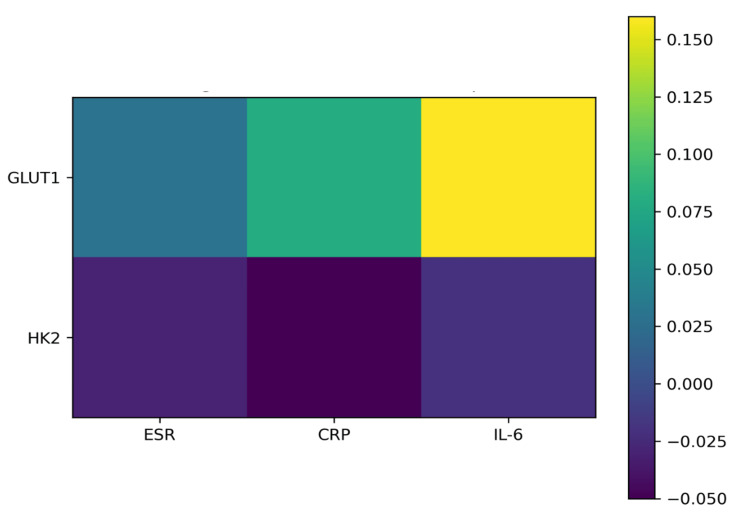
Spearman correlation heatmap

The Spearman correlation heatmap provided an overall visual summary of the correlation matrix and showed no dominant clustering pattern or meaningful association between immunometabolic markers and inflammatory activity (Figure 2).

Overall, these findings suggest that GLUT1 and HK2 were not closely aligned with circulating inflammatory markers or DAS28 score within the case cohort, despite their clear elevation in cases compared with controls.

## Discussion

This secondary analysis revealed a clear immunometabolic pattern in RA with high expression of both GLUT1 and HK2 in the disease when compared to healthy controls. This identification is in line with the more recent understanding of RA as a disorder of the cellular metabolism of both the immune and stromal compartments [1,2]. Upregulation of markers of glucose-handling (biologically relevant to increased glycolytic activity, essential for proliferation, cytokine production, migration, matrix remodeling, and survival in hypoxic synovial conditions) has been observed upon activation of RA FLs by inflammatory cells. Previously, we have observed that glucose-handling markers are upregulated in activated RA FLS with an inflammatory cell partner, which is biologically relevant to its increased glycolytic activity, essential for proliferation, production of cytokines, migration, matrix remodeling, and survival in hypoxic synovial conditions [3,4]. Groups are either separated in the case of the two markers (GLUT1 and HK2), or the separation is greater than can be explained by background metabolic variation, which implies that these markers might be related to metabolic activation of the disease and not just background metabolic variation.

The expression of GLUT1 and HK2 is particularly relevant to the synovial tissue biology. It is now known that FLS not only play a role in the inflammatory process through the action of inflammatory cytokines, but they can also actively participate in the pathogenesis of RA. These cells can transform into an aggressive phenotype that has anti-apoptotic properties, increased invasiveness, excessive production of cytokines, and cartilage and bone destruction [12,13]. As a phenotype, it is driven by glycolytic reprogramming and is directly linked to chronic synovial inflammation [15,16]. In metabolically active cells, glucose is transported into the cells by GLUT1, and HK2 converts glucose within the cells to glucose-6-phosphate and starts the glycolytic metabolism. This sequence helps to identify glucose uptake and intracellular glycolytic commitment as two relevant events to persistent inflammatory synovial activation [16,17].

In the current data set, HK2 appeared to be more useful than GLUT1 for the stratification of disease activity. The absence of significant differences in most inflammatory markers across DAS28 activity categories may reflect limited statistical power, treatment-related suppression of inflammatory biomarkers, biological heterogeneity, and incomplete metadata regarding medication exposure and disease duration. The expression of HK2 was significantly higher in cases of high activity as compared to moderate activity, while GLUT1 expression showed a nonsignificant increase in expression with activity level. This pattern is also consistent with a biological reality because the GLUT1 is mainly indicative of glucose transport, while the capacity of HK2 is situated downstream of glucose transport, which might be more indicative of the sustained glycolytic flux [5,6]. The previous studies also suggested the importance of HK2 in the diagnosis and severity of RA, and this is consistent with the concept of using immunometabolic markers to measure the disease burden in RA [5]. The current findings should be considered exploratory because HK2 differed between categorical disease-activity groups but did not show a significant continuous correlation with DAS28. Therefore, HK2 requires prospective validation before it can be interpreted as a disease-activity biomarker.

The inflammatory marker profile showed that there is a broad inflammatory response in systemic circulation and higher ESR, IL-6, SAA, NLR, and PLR in cases than in controls. These results are in keeping with the accepted biology of RA, where an increase in acute phase responses and hematologic changes is typical of inflammatory disease activity [7,11]. IL-6 participates in signalling the acute phase response in the liver, while SAA is a tool to assess systemic activation of inflammation and can provide more information regarding inflammatory burden in the RA-spectrum disease [8,9]. Innate immune activation, lymphocyte redistribution, and platelet-associated inflammatory signaling [18,19] may be some of the mechanisms captured by NLR and PLR. The higher levels of several inflammatory indices indicated that the pattern of inflammation was generalized and not abnormal levels of one index.

CRP levels were unexpectedly lower in cases than in controls, in contrast to the other inflammatory markers, and therefore should be interpreted with caution. Although ESR, IL-6, and SAA were elevated in cases, CRP was unexpectedly lower than in controls. This discordant finding differs from the typical inflammatory profile observed in RA and may reflect dataset-specific factors such as treatment exposure, assay variability, specimen timing, data-entry issues, or unmeasured confounding. Because assay documentation and treatment information were unavailable, this observation should be interpreted cautiously [7,11]. These could be related to treatment exposures, cohort age/ metabolic differences, variation in assay units, specimen timing, batch variation, and data entry error. The dataset was rechecked, and CRP values and units were confirmed to be recorded as provided in the original dataset; however, the discordant finding remains unexplained because assay-platform information and treatment data were unavailable.

A key limitation of this study is the discordance between CRP and the other inflammatory markers. While ESR, IL-6, SAA, NLR, and PLR were elevated in the RA group, CRP levels were unexpectedly lower than those of controls. Because the present analysis was based on a secondary dataset, the underlying reasons for this discrepancy could not be independently verified and may reflect treatment effects, assay variability, sample collection differences, or dataset-specific factors. Additionally, fasting glucose levels differed significantly between groups, introducing a potential source of metabolic confounding. Consequently, the observed associations between inflammatory markers and glucose-transport-related genes should be interpreted cautiously. These findings should be considered exploratory and hypothesis-generating pending confirmation in prospectively collected cohorts with comprehensive clinical and treatment data.

Weak correlations were observed between the inflammatory markers and GLUT1 and HK2, but these were not statistically significant within the cases. This may indicate that, once the disease has broken out, there is not a strong correlation between immune metabolic activation and blood markers of inflammation. Peripheral markers of glycolysis might reflect a more widespread alteration of synovial activation, which is more likely to be captured at the tissue level, particularly in heterogeneous secondary datasets with varying lengths of disease activity, partial representation of DAS28, and missing treatment data [10,15]. The data show that GLUT1 and HK2 are more discriminative for differentiating cases from controls than for measuring short-interval inflammatory variation.

These results are still explorative and speculative. The use of GLUT1 or HK2 in clinical practice would require a prospective study validating it as a biomarker, standardized assays, complete assessment of disease activity, and treatment changes based on this marker, and comparison with imaging or synovial tissue markers, as well as the assessment of metabolic comorbidities. Additional studies are required to establish whether there is a link between circulating GLUT1 and/or HK2 and synovial tissue metabolism, systemic immune-cell metabolic activation, or a combination of both [17,20].

Limitations and future directions

There are some limitations associated with this study. It was generated from a retrospective secondary de-identified dataset, with no information regarding patient recruitment, treatment history, laboratory methods, or when samples were collected. Because the dataset lacked information on assay platforms, calibration procedures, specimen timing, and laboratory quality-control documentation, methodological variability could not be fully assessed. The amount of disease activity data was incomplete, which limited correlation analysis. An out-of-column serology variable was excluded because of a dataset-structure error that prevented reliable linkage of values to individual participants. The case and control groups differed in age and sex distribution, and adjustment for these imbalances using multivariable modelling was not feasible in this secondary anonymized dataset. Therefore, residual confounding cannot be excluded, and the observed associations should be interpreted cautiously. Hence, the lower CRP level in cases needs to be taken with caution. No adjustment for multiple testing was performed because of the exploratory nature of the study; consequently, the possibility of type I error cannot be excluded.

Standardized recruitment and assays, harmonized assays, predefined assay time points, full clinical metadata, and matched control groups should be used in future prospective studies to validate GLUT1 and HK2. Longitudinal studies are needed to determine whether these markers change with disease activity, treatment response, and disease progression. There should also be a correlation with synovial tissue findings, imaging findings, and validated clinical disease activity measures.

## Conclusions

This study showed that patients with RA had significantly elevated expression levels of GLUT1 and HK2 when compared to healthy controls, along with elevated ESR, IL-6, SAA, NLR, and PLR. The results support an exploratory immunometabolic association in RA rather than confirming clinical biomarker utility. In these cases, there was a significant increase in HK2 expression in the high disease activity group, while GLUT1 had only a nonsignificant increase. However, HK2 did not show a significant continuous correlation with DAS28 or circulating inflammatory markers; therefore, this finding should be interpreted cautiously and considered hypothesis-generating. The within-case correlations with these metabolic markers were weak and nonsignificant, so these markers do not necessarily follow the same pattern as circulating inflammatory markers or the DAS28 score. The interpretation is further limited by the retrospective secondary design, demographic imbalance between groups, unavailable treatment and assay metadata, lack of multivariable adjustment, absence of multiple-testing correction, and the discordant CRP finding. Overall, the expression of GLUT1 and HK2 appears to be exploratory immunometabolic markers in RA, but prospective studies with standardized assays, matched controls, complete clinical metadata, and adjusted analyses are required before any diagnostic, prognostic, or disease-monitoring role can be proposed. It is also important for future studies to clarify whether these markers are involved in local synovial metabolism, systemic immune-cell activation, or both.
